# Epithelial–Mesenchymal Transition: Role in Cancer Progression and the Perspectives of Antitumor Treatment

**DOI:** 10.32607/actanaturae.11010

**Published:** 2020

**Authors:** A. V. Gaponova, S. Rodin, A. A. Mazina, P. V. Volchkov

**Affiliations:** Moscow Institute of Physics and Technology, Dolgoprudny, Moscow Region, 141701 Russia; Department of Medical Biochemistry and Biophysics, Karolinska Institute, Stockholm, 17177 Sweden

**Keywords:** epithelial–mesenchymal transition, cancer, metastasis, resistance to anticancer therapy, cancer stem cells, chemotherapy, immunotherapy

## Abstract

About 90% of all malignant tumors are of epithelial nature. The epithelial tissue is characterized by a close interconnection between cells through cell–cell interactions, as well as a tight connection with the basement membrane, which is responsible for cell polarity. These interactions strictly determine the location of epithelial cells within the body and are seemingly in conflict with the metastatic potential that many cancers possess (the main criteria for highly malignant tumors). Tumor dissemination into vital organs is one of the primary causes of death in patients with cancer. Tumor dissemination is based on the so-called epithelial–mesenchymal transition (EMT), a process when epithelial cells are transformed into mesenchymal cells possessing high mobility and migration potential. More and more studies elucidating the role of the EMT in metastasis and other aspects of tumor progression are published each year, thus forming a promising field of cancer research. In this review, we examine the most recent data on the intracellular and extracellular molecular mechanisms that activate EMT and the role they play in various aspects of tumor progression, such as metastasis, apoptotic resistance, and immune evasion, aspects that have usually been attributed exclusively to cancer stem cells (CSCs). In conclusion, we provide a detailed review of the approved and promising drugs for cancer therapy that target the components of the EMT signaling pathways.

## INTRODUCTION

The epithelial–mesenchymal transition is a physiological process by which epithelial cells attain the properties of mesenchymal cells, both morphologically (changes in cell shape) and physiologically (movement and invasion, global changes in expression profile and metabolism).

Epithelial cells are organized into cell layers that interconnect through cell junctions and are adhered to the basement membrane. Although epithelial cells possess some ability to restructure their shape, their migration in any significant manner is confined to the margins of the epithelial layer. The following types of cell junctions that interconnect epithelial cells are usually differentiated: the so-called adherent junctions, tight junctions based on E-cadherins binding to the actin cytoskeleton, and gap junctions and hemidesmosomes that are linked by cytokeratin-based intermediate filaments.

The key components of epithelial cell junctions are the transmembrane molecules E-cadherin and β-catenin, which bind cadherins to the actin cytoskeleton. In vertebrates, over 100 types of cadherin with varied tissue specificities have been identified [1] due to a large variety of genes synthesizing cadherins and alternative splicing. The junctions between vertebrate epithelial cells are formed by E-cadherin homodimers.

Cadherins are transmembrane proteins consisting of an extracellular, a transmembrane, and cytoplasmic domain. The extracellular calcium-binding site is formed by five domains; the transmembrane region consists of a single chain of glycoprotein repeats. The cytoplasmic region is connected to β-catenin and the p120 protein, which stabilizes cadherin on the cell surface. β-Catenin interconnects the cytoplasmic region of cadherin to α-catenin [2, 3]. The latter is connected to actin of the cytoplasmic skeleton and regulates the assembly of actin filaments by repressing Arp2/3- mediated actin polymerization [4]. Proper functioning of this protein complex ensures intercellular adhesion, as well as coordination of the cytoskeletal dynamics, control over cell layer movement during embryogenesis, and tissue morphogenesis and homeostasis [5, 6].

Unlike epithelial cells, mesenchymal cells and fibroblasts do not have an apical-basal polarity and are fusiform in shape. Although they have regions of focal adhesion to the extracellular matrix, these cells can freely move in three dimensions, passing along and through the collagen networks of the extracellular matrix [7, 8].

The phenomenon of epithelial–mesenchymal transition was first described in the early 1980s in Elizabeth Hay’s laboratory [9, 10], in both embryonic notochord and lens epithelial cells isolated from chicken embryos, and in differentiated chicken lens epithelial cells. Epithelial cells placed in a 3D collagen matrix *in vitro *exhibited morphological changes: they acquired a bipolar fusiform shape with long cellular processes, pseudopodia and filopodia, and they also penetrated the matrix [9].

During EMT, epithelial cells undergo a suppression of E-cadherin and the other genes responsible for the synthesis of the components that create firm adherens junctions. This leads to the loss of cell adhesion and apical-basal polarity, cytoskeleton reorganization, and an increase in cell motility. Suppression of epithelial cell expression occurs in combination with increased expression of transcription factors and the associated mesenchymal genes, such as N-cadherin, vimentin, fibronectin and extracellular matrix metalloproteinases [11, 12, 13]. Changes in the expression profiles of the genes responsible for the formation of the epithelial and mesenchymal phenotypes are considered key characteristics of EMT.

## EMT TYPES

The earliest experiments at Elizabeth Hay’s laboratory that demonstrated the existence of EMT showed that this process is typical of both embryonic and differentiated cells [9]. Despite the similarity of the molecular mechanisms underlying EMT, as well as the overarching result of the process (the formation of motile cells with a mesenchymal phenotype in embryonic and differentiated cells), they play fundamentally different functional roles in the body.

Depending on the biological context, three EMT subtypes are typically distinguished: type I EMT occurs during the embryogenesis [14, 15, 16] and morphogenesis of organs [17, 18, 19], type II EMT is related to the regeneration of injured tissues [20, 21] and pathological fibrosis [22-26], and type III EMT is associated with cancer metastasis.

Type I EMT is the earliest EMT type that initially occurs during implantation, when extragerminal cells of the trophectoderm undergo epithelial–mesenchymal transformation and migrate from the blastocyst body to the uterine endometrium, thus contributing to the formation of the attached placenta [27, 28].

The next EMT-related event to occur after implantation is the formation of the primary mesoderm from the primary ectoderm during gastrulation [29, 30, 31]. EMT is one of the mechanisms of ingression (eviction) of cells inside the blastula wall (the blastoderm or the primitive ectoderm), which is histologically an epithelial layer located inside the blastocoel. The cells migrate to a specific area of the embryo, the so-called primitive streak. During invagination, cells from the primitive streak form the mesoderm and endoderm through EMT [15]. The Wnt/β-catenin signaling pathway underlies the regulation of these processes.

Another important EMT-mediated event during embryogenesis is the formation of the neural crest. The neural crest is a collection of cells secreted from the edges of the neuroectoderm during neural tube closure [32]. The population of precursor neural crest cells possesses a high migration potential over the entire embryo and is involved in the formation of various structures in the body, such as the vegetative ganglia of the nervous system, skin melanocytes, facial skeleton cartilage, adrenal chromaffin cells, and heart valves. Similar to the cells undergoing EMT during gastrulation, future neural crest cells lose their N-cadherin-mediated cell adhesion ability and detach from the neuroepithelium. Basement membrane fragmentation then takes place, causing increased expression of the genes responsible for the formation of the mesenchymal phenotype, increased motility, and subsequent active invasion [33]. The migration of neural crest cells is primarily induced by the bone morphogenetic protein (BMP) pathway and its inhibitor. Furthermore, components of the extracellular matrix (high levels of fibronectin and hyaluronic acid are typical of the areas to which the cells of the future neural crest migrate) are among the most important EMT inducers and regulators during neural crest formation [34].

Type I EMT is involved in the morphogenesis of heart valves and the secondary palate. The anlagen of the mitral and tricuspid valves, as well as the interventricular septum of the heart, forms during TGF-β-mediated epithelial–mesenchymal transition of germinal endothelial cells [35]. Furthermore, recent research has shown the importance of the Wnt signaling pathway and hyaluronic acid to EMT during heart morphogenesis [36]. TGF-β3-regulated EMT in the palatine suture underlies accurate morphogenesis of the facial skeleton, and the formation of the secondary palate in particular. The activated TGF-β3 factors Snail and SIP1 bind to the E-cadherin promoter in conjunction with Smad4, thus repressing its transcription [37].

Unlike type I or III EMT, type II EMT is triggered exclusively by tissue damage and inflammation [38]. Type II EMT is part of the complex process of tissue repair and regeneration, playing an important role in tissue re-epithelization and granulation tissue formation. Re-epithelization is a process in which epidermal keratinocytes become motile, gain a mesenchymal phenotype, and migrate to the wound edges. Proliferation and replenishment of the damaged area then starts and continues until the epithelial cells on the opposite edge of the wound are met. From that point on, further cell migration ceases due to the phenomenon of contact inhibition [39].

Wound healing occurs via two parallel processes: re-epithelialization, and the ongoing remodeling (the formation of granulation tissue performed primarily by myofibroblasts that produce large amounts of extracellular matrix proteins) [40]. Many pathways of myofibroblast formation [41, 42], including those formed during EMT, have been reported [43]. Furthermore, TGF-b1, one of the key EMT inducers that also regulates physiological wound healing, is considered the main motive force of fibrosis [45], partially due to its role in myofibroblast activation [44, 46].

Typically, after the re-epithelization is completed, myofibroblasts undergo apoptosis [44]. Disruption of EMT regulation or pathologically prolonged myofibroblast activity caused by chronic or inflammatory damage leads to fibrosis, impaired function, and, ultimately, destruction of the affected organs.

In addition to TGFβ, growth factors such as FGF, HGF, and EGF are the known EMT inducers involved in wound healing [47]. Slug, a crucial transcription factor for EMT, is also part of re-epithelialization: Slug knockout mice have a lower potential for wound healing [20], being that they are related to the impaired migration of epidermal keratinocytes [48].

Cancer-specific type III EMT has been studied the least. Epithelial cancer cells are highly divergent from normal epithelial cells in terms of their infinite replicative potential and resistance to cell signaling that would otherwise suppress their growth and proliferation, as well as their apoptotic resistance, genomic instability, metabolic deregulation, immune avoidance, and intense angiogenesis [49].

One of the key features of cancer cells is their potential for invasion, migration, and formation of metastatic foci in internal organs [49]. Many studies have focused on the role played by EMT activation in the invasion and metastasis of various cancer types, both *in vivo *and *in vitro *[50-53]. Both the mesenchymal phenotype and EMT marker expression in cancer cells are associated with chemo- [54], radio- [55], and immunotherapy [56] resistance, as well as reduced susceptibility to apoptosis and aging signaling [57,58]. Furthermore, elevated expressions of N-cadherin and vimentin are EMT markers that have been found to assist cancer cells in immune avoidance [59].

Many molecular mechanisms found to be responsible for type III EMT are conservative to the previously described type I and II ones. However, there are some unique features of EMT that are used by cancer for dissemination. The mechanisms inducing EMT in cancer cells remain poorly understood, and their role in cancer progression remains unclear and is subject to dispute. A hypothesis has been put forward that alterations in the expression of EMT markers are simply a consequence of the genomic instability of cancer cells and do not indicate that the cells are preparing to undergo embryogenesis-like EMT [60].

Next, we delve into the features of the intracellular and extracellular molecular mechanisms (the effects of the tumor microenvironment) of EMT, which underlie various aspects of tumor progression. We also discuss in detail their potential as molecular targets for antitumor therapy and markers for early cancer diagnosis.

## MOLECULAR MECHANISMS OF EMT IN THE CONTEXT OF CANCER PROGRESSION (INTRA- AND EXTRACELLULAR SIGNALING)

**Intracellular signaling**

The coordination of intracellular signaling that is crucial to the normal functioning of EMT can be disrupted by deregulatory stimuli originating from an altered cell microenvironment, which enables fibrosis development and cancer progression.

**Fig. 1:**
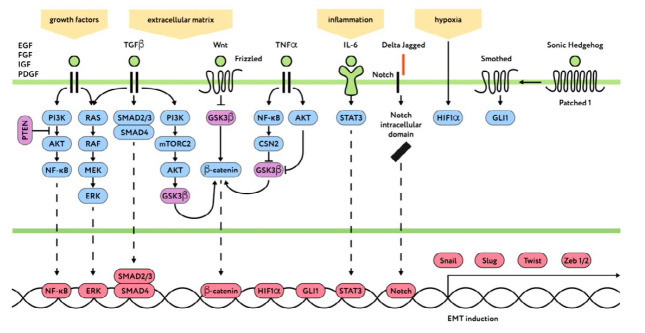
The key signaling pathways that regulate EMT. The components of signal transduction inducing EMT are shown in blue; the components that suppress EMT are shown in violet; transcription factors activating the EMT processes are shown in red

The intracellular signals that regulate EMT are diverse and fairly well understood (*Fig. 1*). The roles played by the following signaling pathways have been described most thoroughly: (TGF)-β/BMP (SMAD-dependent and SMAD-independent variants of this signaling pathway are distinguished in the context of EMT) and Wnt (β-catenin, Notch, and Hedgehog). Additionally, receptor tyrosine kinases such as EGF, FGF, IGF, and PDGF, as well as the key transcription factors (regulated by the previously mentioned pathways and receptors) Snail1, Snail2 (also known as Slug), ZEB1, ZEB2, and Twist, which act as repressors of the E-cadherin expression and other genes responsible for the formation of the epithelial phenotype [61] (*Fig. 1*), have also been described in the literature.

Furthermore, SNAIL and ZEB2 activate the expression of metalloproteinases, which contribute to the degradation of the basement membrane and cancer cell invasion [62].

**Fig. 2:**
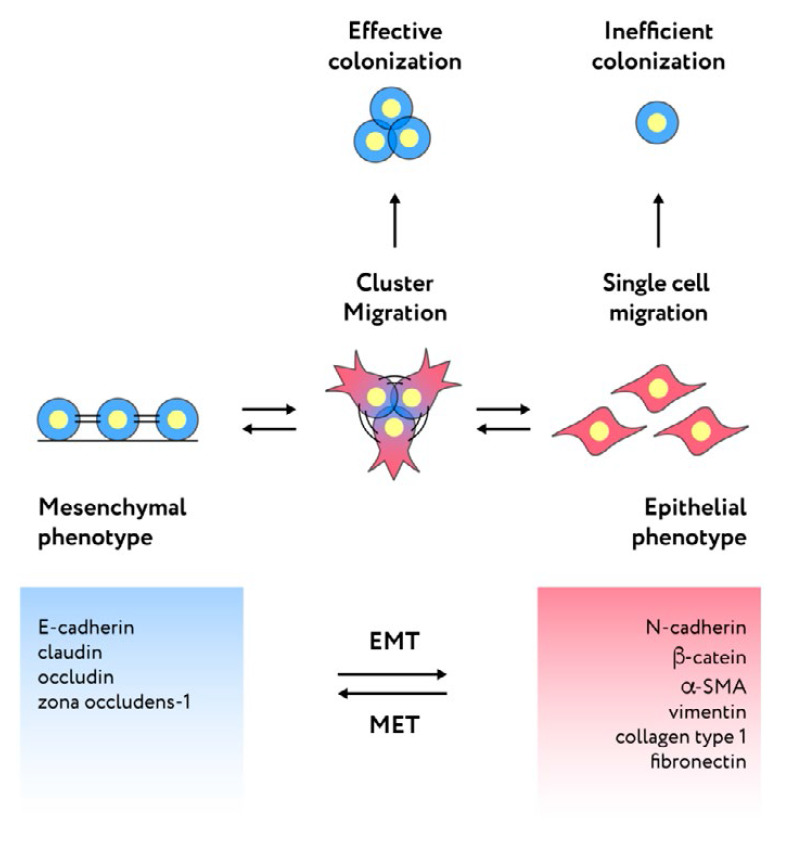
Cell plasticity and the role of the intermediate epithelial–mesenchymal phenotype in the formation of secondary tumor foci (see detailed explanation in the text)

The epigenetic mechanisms of EMT regulation associated with methylation and acetylation of histones and miRNAs are also significant. Activation of the aforementioned molecular mechanisms enables the expression of EMT markers; namely, increased expression of N-cadherin, vimentin, type 1 fibrillar collagen, β-catenin and repression of E-cadherin, claudins, protein zonula occludens 1, occludins, cytokeratins, and matrix activation metalloproteinases (*Fig. 2*).

In pancreatic cancer cells, the transcription factor ZEB1 plays a key role in the regulation of EMT and the metastatic process by suppressing the E-cadherin expression via the recruitment of HDAC1 and HDAC2 deacetylases to the promoter region of the *CDH1 *gene [63, 64]. Suppression of the TGF-β signaling pathway using miR-202 micro-RNA inhibits EMT in pancreatic cancer cells [65].

The transcription factor ETS1, which is characteristically elevated in prostate cancers, activates EMT through the induction of the TGF-β signaling pathway, followed by the activation of ZEB1 and SNAIL1 [66]. Recently, the role of the TRPM4 calcium ion channel in EMT regulation and invasion in prostate cancer cells has been shown to be mediated by the induction of SNAIL expression [67].

The role of the c-Myc proto-oncogene in the induction of EMT and cancer stem cells through the Wnt signaling pathway and activation of ZEB1 in triple-negative breast cancer cells was demonstrated earlier [68]. Additionally, overexpression of miR-93 micro-RNA in breast cancer cells, which suppresses the tumor suppressor PTEN (*Fig. 1*), is associated with EMT and tumor resistance to the cytotoxic activity of doxorubicin [69].

Inhibin B (INHBB), a membrane glycoprotein belonging to the TGF-β superfamily, and the Smad-dependent TGF-β signaling pathway regulate EMT and anoikis in the cells of head and neck squamous cell carcinomas [70]. The TGF-β/Snail and TNF-α/NFκB signaling pathways determine the course of EMT in colorectal cancer [71, 72] (*Fig. 1*). Recently published studies describe the new molecular regulators of EMT involved in the metastasis of lung cancer [73, 74, 75].

**Extracellular Signaling**

due to various stimuli from the local microenvironment, such as growth factors, cytokines, hypoxia, and contact with the surrounding extracellular matrix (the tumor-associated stroma) (*Fig. 1*). Tumor microenvironment factors influence the survival, proliferation, and progression of cancer: that is why they are actively studied.

Inflammation is a critical component of tumor development. Chronic inflammation is associated with an increased risk of cancer. In fact, about 20% of cancers are associated with the chronic inflammation caused by infections, autoimmune reactions, and injury. In addition, the oncogenic signaling pathways in cells susceptible to malignant transformation induce the activation of inflammatory signaling pathways. Thus, tumor tissue infiltration by immune cells and increased expression of proinflammatory cytokines are found in most tumor types regardless of whether an external inflammation is involved in their development or not [76]. A large body of evidence for the role played by various cellular and humoral components of inflammation in the induction of EMT and metastasis has been obtained [77] (*Fig. 1*).

Rapid tumor growth is also associated with a disruption of vascularization, causing the formation of areas of temporary or chronic hypoxia. Hypoxia and activation of hypoxia-inducible factors (HIFs) are observed in many tumors. HIFs regulate the expression of the genes responsible for the survival, proliferation, motility, metabolism, pH regulation, recruitment of inflammatory factors, and angiogenesis processes. Thus, HIF induction promotes cancer progression (as is in the case of fibrosis) and activates EMT and metastasis in many types of cancer [78, 79, 80, 81] (*Fig. 1*).

Laminins are extracellular matrix proteins (to be more specific, heterotrimeric glycoproteins) that constitute the bulk of the basement membrane, which is in direct contact with epithelial cells and ensures proper signal transduction to the cells [82]. Laminins regulate polarization and migration, thereby affecting the epithelial and mesenchymal characteristics of cells during normal ontogenesis and wound healing.

The laminin-111 fragment cleaved by matrix metalloproteinase MMP2 enhances the expression of E-cadherin by suppressing SNAIL 1 and SNAIL2 expression in mouse embryonic stem cells [83].

Mouse mammary epithelial cells are usually subjected to Rac1b-mediated EMT. When treated with matrix metalloproteinase-3 (MMP3), laminin-111 inhibits the transition to a mesenchymal phenotype [84]. Activation of Rac1b (a splice variant of the small GTPase Rac1) mediated by the interaction between laminin-111 and its receptor, α6 integrin, is associated with an increased expression of the keratin-14 epithelial marker and suppression of the mesenchymal markers Snail1, α-smooth, muscle actin, and vimentin. In contrast, fibronectin, another extracellular matrix protein, stimulates EMT in mammary cells through binding to its α5-integrin receptor [84].

The laminin-111 fragment cleaved by the matrix metalloproteinase MMP2 also inhibits tissue fibrosis *in vivo *[85]. *In vitro*, the interaction between this fragment and α3β1 integrin weakens TGF-β1-induced Smad3 phosphorylation and Snail activation in mouse peritoneal cells and inhibits the mesothelial–mesenchymal transition [85], which is a subtype of EMT.

In addition, it has been demonstrated that tumor progression is largely determined by laminins [86]; some isoforms of laminin promote tumor cell migration [87, 88, 89].

Laminins (and laminins within the basement membrane in particular) are the key factor responsible for the attachment and polarity of epithelial cells. Loss of binding and attachment to the basement membrane through laminins is associated with a loss of polarity (one of the first stages of EMT) and also correlates with an unfavorable prognosis of tumor progression [90]. EMT is typically associated with a loss of expression of the basement membrane components [91]: so, certain laminin chains can be regarded as EMT markers.

EMT transcription factors directly affect laminin expression. Snail1 suppresses the α5 and enhances the α4 chain expression of laminin in oral squamous cell carcinoma [92]. ZEB1 inhibits the expression of the α3 chain of laminin and type IV collagen (which also is the primary component of the basement membrane) in colorectal cancer cell lines but increases the expression of laminin γ2-chain [91]. The laminin γ2-chains are known to accumulate in the frontal area of invasive malignant tumors [93] in the form of monomers, rather than as a component of mature laminin trimers or the basement membrane [94].

It was also shown that laminins can directly affect EMT in tumor cells. In hepatocellular carcinoma cells, laminin-332 signaling via integrin-α3 enhances the expression of SNAIL1 and SNAIL2 and inhibits E-cadherin expression [95]. Nevertheless, the involvement of co-stimulatory signals through TGF-β1 is required for EMT completion and transition to the invasive phenotype [95].

Other components of the extracellular matrix, namely fibronectin and collagen, also play an important role in tumor progression. Many studies have indicated that type 1 collagen is related to EMT and invasion. Its isoform, collagen 1A1, is crucial to the progression of non-small cell lung cancer (NSCLC) and is associated with EMT [96]. Progression of gastric cancer also correlates with the expression of type 1 collagen [97]. In addition, collagen fibrils in metastatic lung tumors are characterized by a higher organization as a result of collagen cross-linking with lysyl oxidase (LOX) enzymes. The expression of LOX and LOXL2 lysyl oxidase isoforms is directly regulated by miR-200 and ZEB1, the key regulators of EMT. Stabilization of collagen fibrils due to the activation of lysyl oxidase increases the rigidity of the extracellular matrix and activates the β1/FAK/Src integrin signaling pathway through type1 collagen, thus triggering invasion and metastasis in lung cancer [96]. In a similar way, TGF-β1 induces LOXL2 expression and type 1 collagen stabilization in hepatocellular carcinoma cells, thus promoting invadosome formation and tumor invasion [98].

The increased extracellular matrix stiffness that is due to collagen stabilization induces TWIST-dependent EMT and is a poor prognostic marker for breast cancer [99]. Thus, changes in the physical characteristics of the extracellular matrix, such as stiffness, can initiate EMT processes by mechanical signal transduction to tumor cells, thus promoting invasion and metastasis [99].

Fibronectin, an extracellular matrix component that ensures the connection between collagen fibers and integrin molecules on the cell surface, is also an EMT marker [100]. Fibronectin splicing isoforms containing the ED-B domain are not expressed in normal adult tissue, being present only in the tumor stroma or during embryonic development, which makes them a promising tumor-specific marker of EMT [101].

## CELL PLASTICITY AND CANCER PROGRESSION

As previously discussed, EMT is crucial to a wide variety of body functions at different stages of development in various organs and tissues because of the complex variety of molecular regulatory mechanisms. In a broad sense, EMT ensures one common feature: the so-called cellular plasticity, which is the ability of cells to change their phenotype and function under certain conditions. In addition, cellular plasticity also manifests itself in that cells undergo EMT only partially (*Fig. 2*). Moreover, EMT processes can be reversible. All these processes are required for normal development; the oncogenic mechanisms use the plasticity of the original cell to transform it into a tumor cell in a completely different (pathological) context. Today, there is evidence indicating that partial EMT and the reverse process, mesenchymal–epithelial transition (MET), play a critical role in invasion and metastasis (*Fig. 2*).

In contrast to the complete EMT occurring during embryogenesis, tumor cells usually rarely undergo complete transformation into mesenchymal cells [64, 67, 102-106] but rather form a hybrid epithelial/ mesenchymal phenotype, which manifests itself in the coexpression of both epithelial and mesenchymal markers. Moreover, different cancer types are characterized by different sets of coexpressing markers, which is likely due to variations in the primary pathways involved in progression (see discussion above) (*Fig. 2*).

Surprisingly, certain tumor cell populations retain a high level of expression of E-cadherin, which is crucial in maintaining the epithelial phenotype but interferes with neither the formation of a partial epithelial/mesenchymal phenotype nor its invasive or migratory potential [103, 107-112].

It has been called into question whether metastasis initiation occurs through the EMT mechanism, in experimental studies with transgenic *in vivo *models of breast [113] and pancreatic cancers [114]. However, problems related to the experimental model used by Fischer et al. [113] to study EMT were found later, including the erroneous selection of the *Fspl *and *Vim *genes as mesenchymal markers (low expression in breast cancer cells susceptible to EMT) [115]. Several independent studies have demonstrated the key role played by Snail in the regulation of EMT and metastasis in breast cancer [116, 117]. The conclusions on the non-involvement of EMT in the metastasis of pancreatic cancer drawn based on the significance of Snail and Twist expression in EMT have also been scrutinized [118]. In addition, it has been shown that ZEB1 knockdown in the same transgenic *in vivo *model is associated with a loss of cell plasticity (fixation of the epithelial phenotype by tumor cells), as well as a reduction in the invasive and metastatic abilities [64]. Moreover, it was found in a recent study using a variety of transgenic *in vivo *models that E-cadherin and the p120-catenin expression determine the organotropism of metastatic lesions in pancreatic cancer. Their expression leads to the formation of liver metastases, while not being necessary for lung metastasis formation [112].

A study of tumor material obtained from patients with metastatic breast cancer revealed the important clinical significance of the co-expression of E-cadherin and vimentin: high E-cadherin/positive staining for vimentin, as well as low E-cadherin/ positive staining for vimentin, was associated with the most aggressive triple negative form of the disease. However, the worst prognosis, associated with 10-year non-relapse survival, was associated with a high level of E-cadherin/positive staining vimentin. In addition, a comparison of the expression levels of E-cadherin in primary tumors and the corresponding metastases in the lymph nodes showed that the E-cadherin level is most often unchanged (46% of cases) or increased (43% of cases) in metastases, compared to the primary tumor, being reduced in only 11% of cases [119].

The molecular mechanisms underlying the hybrid epithelial/mesenchymal phenotype are unclear [120] and often difficult to explain solely by the established concept of suppression/activation of transcription of the corresponding “epithelial” and “mesenchymal” genes. In some cases, E-cadherin dysfunction may occur, caused by mutations in the CDH1 gene or associated with aberrant signals of the tumor microenvironment [121], and the dysfunction is not necessarily associated with a decrease in adhesion, but is frequently associated with its increase and constitutive activation, which in some cases is important for metastasis [110].

In a recent study that used a mouse reporter line as an *in vivo *model of pancreatic cancer, Aiello et al. [107] confirmed the possibility that two EMT types are utilized during tumor invasion: complete EMT characterized by reduced E-cadherin transcription and increased vimentin transcription, and partial EMT characterized by the preserved expression of E-cadherin mRNAs and increased vimentin transcription (partial EMT is also characterized by a lower expression of the transcriptional factors Etv1, Prrx1, Zeb1, Twist1, Snai1, Snai2, and Zeb2, compared with complete EMT). Moreover, partial EMT was characteristic of a predominant number of tumors of the mouse model. The predominance of this EMT type was also shown in human breast and colorectal cancer cells. Tumor cells undergoing partial EMT showed no surface staining for E-cadherin during immunocytochemical studies. The authors demonstrated that the mechanisms of partial EMT are associated with recirculation of surface proteins and relocalization of surface E-cadherin to late endosomes [107].

Different EMT programs are associated with different methods of invasion. Tumor cells using partial EMT migrate as multicellular clusters with the preservation of intercellular contacts but can also migrate as single cells; in contrast, during complete EMT invasion and migration they proceed only in the form of single cells [107] (*Fig. 2*). Many studies have confirmed collective migration of tumor cell clusters [64, 109, 110, 122, 123] that undergo partial EMT [106, 123, 124] during invasion.

Although most of the cells forming these clusters express E-cadherin and maintain intercellular contacts, tumor cells at the cluster edges do not express E-cadherin and have a more mesenchymal phenotype. Thus, the “leading” cluster cells undergo completion of EMT to enhance mobility, accompanied by an increased production of the metalloproteinases that destroy the extracellular matrix associated with a renewed expression of E-cadherin, thus contributing to an active invasion of the entire cluster, including its more epithelial cells [105, 107, 110, 111, 125].

It is important to note that metastasis is an ineffective process: only a small fraction of circulating tumor cells avoid elimination and give rise to secondary tumors [126]. Despite the smaller number of circulating clusters of tumor cells compared to single tumor cells, metastases are much more often a result of the colonization of tumor cell clusters [127, 128, 129]. Moreover, these clusters cause the polyclonality of secondary tumor sites [110, 130, 131, 132].

The circulation of tumor cell clusters with partial EMT was discovered in the blood of patients with breast, lung, prostate, and colorectal cancer [124, 133, 134, 135]. It is associated with a poor prognosis: low survival rate, high risk of relapse, and resistance to chemotherapy [130, 136, 137, 138, 139].

Tumor metastasis formation is a multi-stage process and, in addition to invasion, migration, and extravasation (penetration of tumor cells through the blood vessel wall into tissue), includes colonization (proliferation of tumor cells in the secondary tumor site), which is associated with an opposite process, the mesenchymal–epithelial transition, which once again emphasizes the importance of cell plasticity to tumor progression. Metastases are formed by epithelial cells whose morphology is identical to that of primary tumor cells, which is characterized by a re-expression of epithelial markers and repression of EMT factors [51, 106, 140, 141, 142, 143].

Meanwhile, the molecular mechanisms underlying MET have been less studied and are usually associated with the suppression of EMT (*Fig. 2*). MicroRNAs (miRNAs), small non-coding RNAs that regulate target gene expression at the post-transcriptional level, play a significant role in suppressing EMT in various types of cancer [144-151].

However, there are mechanisms that directly stimulate the formation of an epithelial phenotype. Growth differentiation factor-10 (GDF10), also known as bone morphogenetic protein 3B (BMP-3B), inhibits vimentin expression and the migration and invasion of squamous cell carcinoma of the head and neck and increases E-cadherin expression and the sensitivity of tumor cells to cytotoxic therapy through apoptosis induction. The reduced GDF10 expression characteristic of this type of cancer is associated with a decrease in the overall survival rate. Interestingly, GDF10 expression is mediated by SMAD 2/3-dependent activating signals from the type III TGF-β receptor (TGFBR3), whose expression is also reduced in this type of cancer. In addition, GDF10 repression is mediated by signals from ERK, rather than by the classical TGF-β EMT signaling [152].

A component of gap junctions, connexin (namely, its isoform Cx32), stimulates MET in hepatocellular carcinoma cells [153]. Bx32 is a suppressor of hepatocarcinogenesis and metastasis in liver cells, and its expression is reduced in hepatobiliary carcinoma cells compared to normal liver tissue [153]. The mesenchymal phenotype of tumor cells is associated with resistance to apoptosis and cytotoxic chemotherapy, and EMT is considered to be one of the resistance mechanisms. Interestingly, in an article by Yu et al. [153], an obtained line of hepatocellular cancer resistant to the DNA-damaging drug doxorubicin shows signs of EMT; thus, the authors postulated the existence of chemotherapy-induced EMT associated with a reduced expression of E-cadherin and Cx32, as well as increased vimentin expression. Overexpression of Cx32 in doxorubicin-resistant cells induces MET associated with a re-expression of E-cadherin and reduced vimentin expression. However, it is worth noting that the authors somewhat self-confidently declared that there is a role for Cx32 in regulating the sensitivity of tumor cells to chemotherapy and the possibility of using it as a target for therapy based only on the potential relationship between the phenotype and sensitivity, while there were no relevant experiments confirming the sensitization of doxorubicin to cells with Cx32 overexpression [153]. A role for various connexin isoforms in metastasis has also been shown in kidney cancer [154] and melanoma [155].

Another important MET inducer is the GRHL2 transcription factor, which activates the expression of various epithelial adhesion molecules and inhibits the expression of EMT factors, such as ZEB1 [156]. The mechanisms of regulation of tumor progression controlled by GRHL2 are very diverse and obviously depend on tissue type. Moreover, this transcription factor has conflicting effects: it can contribute to tumor progression [157, 158] or suppress tumor growth [159, 160]. A large-scale study of various types of cancer compared to normal tissue samples revealed the complex expression patterns of GRHL2, being indicative of both a reduced and increased expression in various tumors. Interestingly, increased expression was observed in proliferating epithelial cells with stem cell characteristics. This was also confirmed in a study focused on the role of GRHL2 in pancreatic cancer [157] and squamous cell carcinoma of the head and neck [161]), as well as in non-invasive types of cancer [159]. In addition, increased expression of GRHL2 is associated with increased proliferative activity, large tumor sizes, and late clinical stages of colorectal cancer. GRHL2 negative breast cancer is quite rare but is commonly associated with metastasis of the lymph nodes. Meanwhile, overexpression in breast cancer cells stimulates proliferation and is associated with the lowest rate of disease-free survival [162, 163]. A similar dual effect of GRHL2 is observed in prostate cancer [164]. Kidney and stomach cancers are characterized by a high frequency of GRHL2-negative tumors [159]. In these types of cancer, it acts as a cancer suppressor and inhibits invasion and metastasis [165, 166].

The role of reprogramming factors in the induction of MET and their impact on tumor progression is poorly understood. It was shown that during the production of induced pluripotent stem cells (iPSCs) from murine fibroblasts by induction of the overexpression of the reprogramming factors Oct3/4, Klf4, c-Myc, and Sox2 (OKMS), the epithelial program associated with the induction of the expression of miR- 205/miR-200 and suppression of Snail1 and TGF-β1/ TGF-βR2 is activated, while the cells undergo MET [167, 168].

Tumor cell reprogramming experiments exert rather conflicting effects on malignant progression. On the one hand, reprogramming leads to a loss of oncogenicity [169, 170] and the suppression of metastasis [171, 172, 173], which is associated with MET, while, on the other hand, the expression of reprogramming factors is associated with a poor disease prognosis [172, 174, 175, 176]. Thus, induction of EMT using reprogramming factors and the potential of this approach as potential antitumor therapy requires further studies and a deeper understanding of the molecular mechanisms underlying the relationship between pluripotency and cell plasticity.

The initiation of MET at the stage of tumor cell colonization of foreign tissues during metastasis is associated with changes in the microenvironment, the absence of external EMT-inducing stimuli from the tumor-associated stroma, and changes in the level of oxygenation of the surrounding tissue [177-180].

## EMT AND RESISTANCE TO ANTITUMOR THERAPY: ROLE IN THE FORMATION OF TUMOR STEM CELLS

**Chemotherapy**

For many cancer types, epithelial–mesenchymal transition is associated with a poor prognosis not only in relation to metastasis. EMT is one of the mechanisms underlying the development of resistance to the cytotoxic effect of antitumor drugs, which is the main challenge in modern oncology. Moreover, while the need for EMT for metastasis was called into question for pancreatic and breast cancer as discussed previously, its role in the development of resistance to chemotherapeutic drugs is not controversial [113, 114].

Overexpression of miR-93 micro RNA induces EMT and reduces sensitivity to the cytotoxic effects of doxorubicin in breast cancer cells. In addition, the gene expression levels associated with multidrug resistance were significantly increased in MCF-7 cells, with miR-93 overexpressed compared to the control. It had been previously shown that miR-93 interacts with PTEN mRNA, a known regulator of EMT in breast cancer cells [69]. Another micro RNA suppressing PTEN expression, miR-21, is also involved in EMT induction and the development of gemcitabine resistance in breast cancer cells [181].

The transcription regulator induces eIF4E Snail expression and triggers the EMT associated with invasion and resistance to cisplatin in nasopharyngeal carcinoma cells [182]. In glioblastoma cells, STAT3 activates the expression of Snail1, causing tumor resistance to another cytostatic drug, temozolomide. The use of antibodies blocking IL-6 prevents STAT3 activation and Snail expression, thus increasing the sensitivity of glioblastoma cells to temozolomide in combination therapy [183].

STAT3 activation due to Y705 phosphorylation in ovarian cancer leads to EMT induction and the development of tolerance to cisplatin. This activation of EMT is associated not with Snail, but rather with another transcription factor important for the formation of the mesenchymal phenotype Slug [184]. In addition, the authors attributed the development of cisplatin resistance directly to a decrease in autophagy caused by STAT3 activation; however, it is worth noting that the direct role of Slug activation in this study was not evaluated [184]. Meanwhile, many research groups have confirmed the direct role of Snail and Slug in the development of resistance to chemotherapy and radiotherapy in ovarian cancer [55, 185-188]. Increased Slug activation is associated with resistance to radiotherapy and temozolomide treatment in patients with malignant glioma. Patients with lower levels of Slug expression demonstrate longer progression-free survival [189]. A role for Slug in the development of multidrug resistance in the MCF-7 breast cancer cell line has also been shown. Slug induces the expression of MMP1 metalloproteinase by directly binding to the promoter region of the gene. A high level of MMP1 is associated with rapid progression and metastasis, as well as poor prognosis in patients with breast cancer [190].

Tumor suppressor FBXW7 triggering ubiquitin-dependent degradation of many oncogenic factors such as Myc, c-Jun, Cyclin E, and Notch1 is responsible for the degradation of Snai1 in non-small cell lung cancer cells. FBXW7 overexpression suppresses NSCLC tumor progression by arresting the cell cycle, inhibiting EMT, and increasing the sensitivity to chemotherapy. Tumor samples obtained from patients with NSCLC are characterized by reduced FBXW7 expression in most NSCLC tissues; the reduced expression level correlates with a later stage of the disease according to TNM staging and worse 5-year survival rate [191].

The use of chemotherapeutic drugs is well studied, being one of the most common approaches to cancer therapy. The cytotoxic effect of these drugs (as well as radiotherapy) extends mainly to rapidly dividing cells, since their mechanism of action involves various types of DNA damage and disruption of mitotic spindle formation. Thus, cells with a mesenchymal phenotype characterized by a lower proliferation index are less sensitive to the cytotoxic effect of chemotherapy compared to those with an epithelial phenotype [75, 106, 192, 193]. In addition, several recent studies have demonstrated the direct effect of EMT on the well-known mechanisms of tumor cell tolerance to massive DNA damage associated with DNA repair [194, 195, 196], cell-cycle control [197, 198, 199], inactivation of reactive oxygen species [200, 201], and autophagy [202]. Thus, the molecular mechanisms behind the development of resistance to chemotherapy are diverse and, for many types of cancer, mediated by the launch of EMT; however, their relationship remains poorly understood.

**Targeted antitumor therapy**

Understanding of the contribution made by EMT to malignant progression has changed significantly since its discovery. Today, it is obvious that EMT plays roles other than those of the formation of a mesenchymal phenotype for tumor cells capable of invasion and migration. The EMT mechanisms can directly affect the triggering oncogenic mechanisms. Unlike cytotoxic chemotherapy, targeted antitumor therapy is aimed at specific molecular targets: proteins specific to a particular cancer type that trigger and promote tumor growth. EMT underlies the development of resistance to targeted drugs in some types of cancer. The role of EMT in the development of resistance to targeted therapy in lung cancer has been described in the greatest detail.

According to the American Institute for Cancer Research (AICR), lung cancer was the most common cancer in the world among all cases documented in 2018. Non-small cell lung cancer (NSCLC) accounts for most (about 85%) lung cancers. Activating mutations in the epidermal growth factor receptor (EGFR) gene are found in 40–89% of NSCLCs. These mutations increase the activity of the intracellular signaling pathways through autophosphorylation of the cytoplasmic section of EGFR receptor tyrosine kinase, leading to the induction of a proliferation of lung tissue epithelial cells, increased angiogenesis, invasion, and metastasis [203]. Targeted therapy aimed at inhibiting the activity of EGFR by drugs such as gefitinib, erlotinib, and afatinib is the basis for treating patients with activating mutations in the EGFR gene. However, as for cancer chemotherapy, the main challenge standing in the way of long-term effectiveness is the initial and acquired tumor resistance to the mechanism of action of an inhibitor. Various attempts have been made to solve this issue, including those related to the suppression of the EMT mechanisms.

Overexpression of TWIST1, one of the key transcription factors in EMT, has been shown to cause EGFR mutant NSCLC cells to become resistant to the EGFR inhibitors erlotinib and osimertinib [204]. Osimertinib is a third-generation EGFR inhibitor approved in 2017 for the treatment of NSCLC in patients with a specific EGFR T790M mutation that either exists *de novo *or is acquired during treatment with first-line drugs (gefitinib, erlotinib or afatinib) and is associated with resistance to these drugs. However, resistance to the antitumor effect of osimertinib occurs within approximately 10 months after treatment and is associated with the onset of the C797S mutation in EGFR exon 20. It is important to note that there is currently no approved pharmacological treatment for EGFR mutant NSCLC that progresses after the development of resistance to osimertinib. Inhibition of TWIST1 activity using an inhibitor in erlotinib- and osimertinib-resistant NSCLC cells increases their sensitivity to the cytotoxic effect of EGFR inhibitors in a dose-dependent manner. Moreover, the sensitization mechanism is associated with TWIST1 suppressing the transcription of proapoptotic BCL2L11 (BIM) by binding to the promoter region of the gene [204].

In addition, erlotinib-resistant NSCLC cell lines exhibit a mesenchymal phenotype (decreased E-cadherin expression and induction of vimentin and N-cadherin) and are characterized by the activation of not only TWIST1, but also Snail, Slug, and ZEB1. Moreover, overcoming of resistance to erlotinib with furamidine, a PRMT-1 inhibitor, was associated with EMT suppression and restoration of epithelial characteristics [205]. A number of studies have also confirmed the role played by EMT in the development of gefitinib resistance and the reversibility of resistance as a result of MET [206, 207].

In 3–7% of cases, NSCLC is associated with various translocations in the ALK gene, leading to the formation of more than 19 chimeric proteins, including EML4, KIF5B, KLC1, and TPR. However, regardless of the genes involved in the translocation, all chimeric products retain the ALK kinase domain, which is responsible for constitutive oncogenic activation of the ALK signaling pathways (including Ras/Raf/MEK/ERK1/2, JAK/STAT, PI3K/Akt, PLC-γ signaling pathways) that regulate migration, proliferation, and cell survival [208]. Most chimeric ALKs are susceptible to the inhibitor crizotinib, which has been shown to be highly effective in the treatment of similar forms of NSCLC. However, resistance to crizotinib treatment develops in most patients within a few years.

It has been found that some NSCLC lines (H2228 and DFCI032, but not H3122) with oncogenic activa tion of ALK express low E-cadherin levels and high levels of vimentin and other mesenchymal markers. Additionally, ALK inhibition changes the cell phenotype to an epithelial one [209]. In a recent paper by Nakamichi et al. [210], H2228 lines resistant to three different ALK inhibitors (crizotinib, alectinib, and ceritinib) were created. The obtained stable line was characterized by a reduced ALK expression and overexpression of another oncogenic protein, AXL, which is associated with EMT and stem cells. Moreover, the artificial induction of EMT using TGF-β1 was also associated with increased AXL expression. The AXL inhibitor was of assistance in the detection of cells resistant to ALK inhibitors [210]. Hence, AXL activation can be regarded as the mechanism underlying tumor resistance to ALK inhibitors. It also induces EMT when ALK expression is low. It is EMT that is responsible for the development of the resistance. Blocking it at the AXL level, in conjunction with HDAC inhibitors, overcomes the resistance of NSCLCs with mutant ALK [211]. Long-term administration of sunitinib to treat kidney cancer also causes the activation of AXL and EMT [212].

Recent studies have also shown that EMT associated with methylation of the E-cadherin gene underlies the development of resistance to hormone therapy with tamoxifen in estrogen-positive breast cancer [213]. In HER2 positive cancer, EMT plays a key role in the development of resistance to the targeted drug trastuzumab [214, 215].

**Immunotherapy**

Antitumor immunotherapy aims to activate immune cells to recognize and induce cytotoxicity in tumor cells. Inhibitors of immune checkpoints (namely, CTLA4, PD-1, and PD-L1 inhibitors) are currently among the main and most successful forms of cancer immunotherapy. In 2018, the researchers James P. Allison and Tasuku Honjo were awarded the Nobel Prize in medicine and physiology for discovering this therapeutic approach and the molecular mechanisms underlying it.

CTLA4 is expressed on the surface of activated T cells (as well as on the surface of regulatory T cells (Tregs)) and interacts with the CD80 and CD86 molecules on the surface of antigen-presenting cells. Unlike the homologous co-stimulatory molecule CD28 (which also binds to CD80 and CD86), CTLA4 is a co-inhibitor of the T-cell receptor signal response and suppresses the immune response, thus maintaining the balance and preventing the development of autoimmune processes [216]. James P. Allison et al. were the first to show that the use of antibodies blocking CTLA4 enhances the immune response against tumors and causes their rejection *in vivo *[217]. Identically to CTLA4, the PD-1 membrane protein suppresses the immune response. PD-1 expressed on the surface of T lymphocytes interacts with PD-L1 and PD-L2 molecules, which are normally expressed on the surface of antigen-presenting cells. In addition, tumor cells use the expression of PD-L1 on their surface to dodge the immune response [218, 219]. Honjo et al. demonstrated that inhibition of PD-1 activates the antitumor immune response regardless of the PD-L1/PD-L2 status of the tumor, while causing a milder autoimmune effect compared to the inhibition of CTLA-4 [220].

Various inhibitors of CTLA4, PD-1, PD-L1, and combinations thereof are now approved for the treatment of melanoma, renal carcinoma, non-small cell lung cancer, squamous cell carcinoma of the head and neck, urothelial carcinoma, colorectal cancer, and Hodgkin’s lymphoma. Moreover, these inhibitors are used both as adjuvant therapy and as second- and third-line therapy when chemotherapy and targeted anticancer drugs fail due to the emergence of resistance. An exception is the metastatic form of non-small cell lung carcinoma with a high level of PD-L1 expression and wild-type EGFR and ALK, which require a combination therapy with ipilimumab and nivolumab (CTLA-4 and PD-1 inhibitors, respectively) as first-line treatment [221]. Today, immunotherapy is the last therapeutic option for many cancer patients in the case when chemo- and targeted therapy are ineffective.

It was discovered that EMT is associated with an increased expression of PD-L1 [222-227], as well as CD47, an inhibitory surface protein blocking phagocytosis [228] in tumor cells and hiding them from immunological surveillance (in particular during invasion and migration to secondary organs, resulting in metastasis formation). Moreover, in NSCLC, EMT is associated with reduced CD4/CD8 infiltration by T lymphocytes, which play a key role in the antitumor immune response [229] and increase the immune response. Additionally, the EMT is associated with suppression of CD4/Foxp3 T-regulatory lymphocytes [230]. Expression of EMT markers in NSCLC tissues is associated with an increased expression of the immune checkpoints PD-L1, PD-L2, PD-1, TIM-3, B7-H3, BTLA, and CTLA-4 [230] and the expression of immunosuppressive cytokines such as IL-10 and TGF-β; however, the underlying molecular mechanisms remain unclear [229].

Tumors characterized by a high level of T-lymphocyte infiltration can be expected to be more sensitive to PD-1/PD-L1 inhibitors. However, a large number of patients with this type of tumors do not respond to such therapy. Using data from the tumor expression profile database (The Cancer Genome Atlas (TCGA)), Wang et al. found a positive correlation between the expression of EMT markers and the level of T-lymphocyte infiltration in urothelial tumors. However, in a study of a group of patients with urothelial cancer treated with nivolumab (PD-1 inhibitor), it was shown that the high level of expression of EMT markers in tumors with a high level of T-lymphocyte infiltration was associated with a poor response to therapy and lower survival rate. Interestingly, tumor stromal cells act as a source of increased expression of EMT markers [231].

The development of tumor resistance to therapy with immune checkpoint inhibitors has been little studied thus far. Some studies indicate that EMT may be involved in this process; however, further research is needed to understand the exact molecular mechanisms.

**Cancer stem cells**

Currently, the classic concept explaining the development of resistance to antitumor therapy is rooted in the presence of cancer stem cells (CSCs). CSCs express markers characteristic of normal stem cells, for example CD44, CD133, CD34, and EpCAM. Through many different mechanisms, CSCs become resistant to chemotherapy and radiotherapy (unlike most of the differentiated tumor cells that undergo apoptosis in the case of effective therapy) [232, 233, 234], migration (abundant data indicate the role of CSC in metastasis [235]), and most importantly, subsequent division and differentiation into different lines of tumor cells, thus ensuring the heterogeneity of the recurrent tumor and the emergence of clones resistant to the therapy used [236].

Although CSCs undoubtedly possess the characteristics inherent to normal stem cells, there is no clear understanding of their origin. This is due to the challenges related to identifying stem markers that may differ in various types of tumors. It is likely that the same reason is behind why CSCs have not been identified for all cancer types [237]. Furthermore, it is very likely that the CSCs in these cancers have different origins.

There are several theories regarding the possible origins of CSCs. According to the first one, CSCs form from the stem cells of mature tissue, ensuring its renewal as a result of somatic mutations. It was shown that CSCs initiating acute myeloid leukemia are not only capable of differentiating into all types of blood cells but can also retain a potential for self-renewal and restoration of hematopoiesis in a series of transplantations in irradiated mice, which is the main characteristic of hematopoietic stem cells. This fact suggests that in the case of leukemia, CSCs arise from hematopoietic stem cells as a result of mutations, which enables the tumor cell to utilize stem regulatory signaling pathways to advance tumor progression [238].

The second theory involves the formation of CSCs from differentiated cells by dedifferentiation and gain of stem cell characteristics. This assumption is rooted in an understanding of cell plasticity and the possibility of reprogramming somatic cells into pluripotent stem cells [239]. Moreover, a recent study on prostate cancer lines has shown that such reprogramming is possible and can be induced by the development of resistance to therapy [240].

To date, the specific molecular mechanisms underlying the reprogramming of tumor cells into CSCs remain poorly studied; however, there is reason to believe that these mechanisms are associated with EMT. EMT activation by ectopic expression of Snail or Twist, as well as by activation of TGF-β1 in an epithelial cell line of breast cancer, is associated with the induction of stem marker expression (the appearance of CD44+/CD24- cells) and their increased ability to form “mammospheres” (tumor-like structures, each being a clone of a single CSC) [241]. Moreover, EMT activation via the Ras-MAPK signaling pathway in normal breast CD44-/CD24+ cells leads to their transformation into CD44+/CD24- stem tumor cells; additional activation of TGF-β1 enhances the effect [242]. A recent study on transgenic mouse models of breast cancer, MMTV-PyMT, showed that although CSCs and normal breast stem cells are phenotypically similar, they form in different parts of the breast epithelium (luminal and basal epithelial regions, respectively) and also differ in terms of the molecular mechanisms of EMT activation (using the transcription factors Snail and Slug, respectively). This study supports the theory according to which CSCs originate from differentiated cells by being reprogrammed during EMT [125]. A role for EMT in the formation of CSCs and resistance to antitumor therapy and the metastatic progression associated with these processes has also been shown in pancreatic cancer [243, 244], prostate cancer [245], squamous cell carcinoma of the head and neck [158, 246, 247], stomach cancer [248, 249], melanoma [250], glioblastoma [251], and colorectal cancer [252, 253].

## CONCLUSION: EMT PATHWAYS ARE MOLECULAR TARGETS FOR ANTITUMOR THERAPY

In this review, we have examined the role of EMT mechanisms in tumor progression, as well as the latest experimental and clinical data confirming the involvement of EMT in almost all of its aspects: tumor invasion and metastasis, resistance to cytotoxic and targeted therapy, and avoidance of immune surveillance. In our opinion, the most crucial aspect is the potential contribution of EMT to the emergence of CSCs, which is the basis of tumor heterogeneity according to modern theories. It is one of the primary roadblocks to cancer treatment and also a key factor in relapse. Thus, the genes within the signaling pathways and direct transcription factors that activate EMT become promising molecular targets for antitumor therapy. These are usually inhibitors of the key components of oncogenic signaling pathways that regulate not only EMT, but also proliferation, growth, survival, and angiogenesis. Moreover, the therapeutic efficacy associated with inhibiting a specific protein associated with EMT depends on the tumor type, since, as has been discussed above, different signaling pathways in EMT regulation can be utilized during tumor progression depending on the tissue type.

**Table:** Antitumor drugs suppressing various components of the EMF signaling pathways (see detailed explanation in the text)

Drug	Target	Clinical trials	Disease
Vismodegib	Smoothened (Shh signaling pathway)	Approved	Metastatic, inoperable, radiotherapy-resistant form of basal cell carcinoma
Temsirolimus and everolimus	mTOR (PI3K/AKT/mTOR signaling pathway)	Approved	Renal carcinoma, relapse of lymphoma resistant to other types of therapy, chronic lymphocytic leukemia
Galunisertib	TGFβRI	Phase 1 Phase 2 and 3	metastatic form of pancreatic cancer, myelodysplastic syndrome
Fresolimumab	TGFβ	Phase 2	Metastatic breast cancer, melanoma, kidney carcinoma, malignant pleural mesothelioma, non-small cell lung carcinoma
Tarextumab	Notch	Phase 1b/2	Stage IV pancreatic cancer
Vantictumab	Frizzled	Phase 1	Stage IV pancreatic cancer, NSCLC, metastatic breast cancer
Harmine	TWIST1	Preclinical evaluation	NSCLC

There are already approved drugs for combinational therapy (used in combination with tyrosine kinase inhibitors or other chemo- and radiotherapy agents) and even some that can be used as monotherapy if there are no other therapeutic options, as well as second- and third-line therapy in patients who have developed drug resistance (*Table*).

An inhibitor of the canonical Shh signaling pathway, the smoothened receptor inhibitor vismodegib, has been approved for the treatment of the most common form of skin cancer, basal cell carcinoma (metastatic and inoperable disease forms), or in cases of relapse after surgical treatment and radiotherapy [254]. Inhibitors of the PI3K/AKT/mTOR components of the EMT signaling pathway, cell cycle, and VEGF signaling have been approved for the treatment of kidney carcinoma (mTOR inhibitors temsirolimus and everolimus) [255], relapses of lymphoma resistant to other types of therapy, and chronic lymphocytic leukemia, in combination with rituximab (idelalisib, a PI3K inhibitor) [256]. Furthermore, a number of inhibitors are currently undergoing clinical trials, mainly in combination therapies. Clinical trials (phase 1) of the TGFβRI inhibitor galunisertib in combination with the PD-L inhibitor durvalumab in patients with metastatic pancreatic cancer (NCT02734160) and as monotherapy in patients with advanced cancer that has spread to other body parts (NCT01373164) have been completed; clinical trials to evaluate its combination with gemcitabine in patients with an unresectable metastatic disease form are currently in phases 1 and 2 (NCT02154646). The data from the latest study have been published and have confirmed the benefits of combination therapy compared to chemotherapy with gemcitabine. In addition, potential predictive markers of sensitivity to the therapy were determined by analyzing tumor samples derived from the patients [257]. Galunisertib was tested in phase 2 and 3 trials in patients with myelodysplastic syndrome of varying severity (NCT02008318). This treatment had an acceptable safety profile and was associated with hematological improvements in patients with low and medium risks of transformation into acute leukemia, and a positive response in patients with signs of an early stem cell differentiation blockage. Many clinical trials seeking to evaluate galunisertib for the treatment of various types of tumors have been initiated in various therapeutic regimens (clinicaltrials. gov).

Fresolimumab, a monoclonal antibody that binds all isoforms of the transforming growth factor TGF-β, in combination with radiotherapy, has completed phase 2 clinical trials in the treatment of patients with metastatic breast cancer (NCT01401062). Molecular markers of sensitivity to fresolimumab therapy have been identified, and the potential for using it in combination therapy with PD-1 blockade in order to enhance effectiveness was assessed. In addition, the drug is being tested in patients with melanoma and renal carcinoma (NCT00356460), malignant pleural mesothelioma (NCT01112293), and non-small cell lung carcinoma, in combination with radiotherapy (NCT02581787).

The Notch inhibitor tarextumab, which has been shown to be effective in preclinical trials, failed in phase 1b/2 of a randomized clinical trial set to evaluate a combination therapy (in combination with etoposide and platinum drugs) for small cell lung carcinoma (NCT01859741). The drug has also been tested in combination with nab-paclitaxel and gemcitabine for the treatment of patients with treatment-naïve stage 4 pancreatic cancer (NCT01647828). The Frizzled inhibitor vantictumab (NCT02005315) has also been used in a study with a similar design. In addition, vantictumab has successfully concluded phase 1 trials in the treatment of patients with NSCLC (NCT01957007) and metastatic breast cancer (NCT01973309).

A TWIST1 inhibitor, alkaloid harmine, causing the degradation of TWIST1 homodimers and TWIST1- E2A heterodimers is currently in the preclinical stage of trials. Harmine per se was shown to have a cytotoxic effect on a NSCLC line with mutated EGFR, Kras, and c-Met. It also proved effective in *in vivo *models, both in transgenic mice with a KRAS mutation and in xenograft models derived from patient tumor tissue (PDX – patient-derived xenograft). Thus, harmine is a promising targeted antitumor drug to be used both in NSCLC monotherapy and as a third-line drug for patients resistant to EGFR inhibitors, which is the regimen that will most likely be tested during the clinical trials.

The molecular mechanisms of EMT regulation are a promising research field in antitumor therapeutics. It is important to use our scientific knowledge about EMT both in our efforts to create new therapies and in order to improve the existing ones. Pharmacological suppression of EMT can help not only to limit metastasis development and overcome resistance to existing therapies, but also to suppress CSCs, the culprit in tumor recurrence. In some cases, drugs that inhibit the EMT are the only available therapeutic option when other types of therapy are ineffective.

## Abbreviations

EMT: epithelial–mesenchymal transition
MET: mesenchymal–epithelial transition
iPSCs: induced pluripotent stem cells
NSCLC: non-small cell lung cancer
CSCs: cancer stem cells
